# Acute Rheumatic Fever in an Adult Female Patient Presenting With Chest Pain: A Case Report

**DOI:** 10.7759/cureus.80133

**Published:** 2025-03-06

**Authors:** Morgan Reel, Jason Haidar, Alexandra Blanco, Eric Boccio

**Affiliations:** 1 Emergency Medicine, Memorial Healthcare System, Hollywood, USA; 2 Emergency Medicine, Florida International University, Herbert Wertheim College of Medicine, Miami, USA

**Keywords:** acute rheumatic fever, carditis, case report, revised jones criteria, rheumatic heart disease, valvulitis

## Abstract

Acute rheumatic fever (ARF) is an autoimmune disease triggered by group A *Streptococcus* (GAS) infection, predominantly affecting children in low- and middle-income countries. Although its incidence has significantly decreased in the United States (US), ARF remains a major cause of morbidity and mortality worldwide due to complications like rheumatic heart disease (RHD). The condition arises from molecular mimicry, where the immune system targets host tissues after a GAS infection, leading to inflammation and potential damage to the heart, joints, and nervous system. Early diagnosis and treatment, including antibiotics and anti-inflammatory drugs, are crucial to managing ARF and preventing recurrent episodes and long-term complications like RHD.

A 39-year-old female patient with a history of recurrent streptococcal pharyngitis and allergy to penicillin presented with three days of fever, sore throat, and left wrist pain and one day of chest pain. Physical examination revealed pharyngeal erythema and tonsillar exudates with no abnormal heart sounds or subcutaneous nodules. Laboratory tests confirmed a positive rapid strep test, elevated inflammatory markers, and an elevated troponin-I level, suggesting carditis. Given the clinical findings, including two major and two minor Jones criteria, the patient was diagnosed with ARF and administered azithromycin, naproxen, and dexamethasone in the emergency department. An inpatient echocardiogram revealed no significant valvular disease, and the patient was discharged on a 10-year course of prophylactic azithromycin.

While ARF remains a significant public health issue affecting children in developing countries and is driven by socioeconomic factors and inadequate healthcare access, it is much rarer in adult patients residing in the continental US. The pathogenesis involves an autoimmune response to GAS, leading to multisystem involvement. Diagnosis relies on assessing the presence of clinical revised Jones criteria, and management focuses on treating the acute infection, controlling inflammation, and preventing disease recurrence and progression through long-term antibiotic prophylaxis. Patients with suspected carditis should be further evaluated for RHD. Continued research and improved healthcare strategies are essential to reduce the global burden of ARF and RHD.

## Introduction

Acute rheumatic fever (ARF) is an autoimmune disease that follows infection with group A *Streptococcus* (GAS). The epidemiology of ARF shows a significant burden in low- and middle-income countries, with an estimated 20 million people affected globally, and primarily affects children [1,2]. ARF is no longer considered a notifiable disease in the continental United States (US), and its estimated annual incidence in 2010 declined to approximately 14 cases per 100,000 in school-aged children and seven cases per 100,000 in adults [3]. ARF is closely associated with low socioeconomic status, overcrowding, and limited access to healthcare. These conditions facilitate the spread of GAS and hinder the timely treatment of streptococcal pharyngitis [4]. The pathogenesis of ARF involves molecular mimicry, where antibodies generated against GAS antigens cross-react with host tissues, particularly in the heart, joints, skin, and central nervous system. The autoimmune response leads to inflammation and subsequent tissue damage and may manifest as carditis, valvulitis, arthritis, chorea, subcutaneous nodules, and erythema marginatum [5]. Carditis and valvulitis resulting in damage to the heart valves are the most severe acquired manifestations of ARF, which may progress to rheumatic heart disease (RHD) [5]. The diagnosis of ARF is clinical and requires evidence of a preceding GAS infection and the presence of revised Jones criteria consisting of major (carditis, polyarthritis, chorea, erythema marginatum, and subcutaneous nodules) and minor (fever, arthralgia, elevated acute phase reactants, and prolonged PR interval) criteria [6]. Management of ARF involves treating the acute infection, reducing inflammation, and preventing recurrence. The primary treatment for acute GAS infection consists of antibiotics, and penicillin is considered the first line in the absence of any known allergies. Anti-inflammatory drugs, typically aspirin or corticosteroids, are used to manage arthritis and carditis [7,8]. Long-term prophylactic treatment is crucial to prevent recurrent episodes of ARF and its progression to RHD [9].

We discuss an unusual case of ARF in an adult patient who resided in the US and presented to the emergency department with fever, sore throat, left wrist arthralgia, and chest pain. The workup revealed a positive rapid strep test and an elevated troponin-I level. The patient was diagnosed with ARF and subsequently treated with antibiotics and anti-inflammatory drugs. Due to the clinical suspicion of carditis and the risk of disease recurrence and progression to RHD, the patient was admitted to the hospital for further management.

## Case presentation

A 39-year-old female patient with a past medical history of recurrent streptococcal pharyngitis infections presented to the emergency department with three days of fever, sore throat, and left wrist pain and one day of chest pain. The patient was seen by her primary care provider via a telehealth visit two days earlier and was prescribed cephalexin 500 milligrams (mg) per os (PO) three times daily (TID). The patient reported a sore throat, muffled voice, and dysphagia. The left wrist demonstrated mild swelling but no erythema or hyperemia. The pain was mild and intermittent, and the patient denied any known trauma or overuse injury. The patient experienced an episode of substernal chest pressure, mild in severity and non-radiating, lasting approximately seven minutes in duration on the morning of the presentation. There were no exacerbating or alleviating factors. She also reported a pruritic rash on her right upper eyelid, which prompted her to seek medical attention as she thought it may be secondary to an allergic reaction to the antibiotic. The patient denied visual changes, headache, cough, and shortness of breath. The patient reported a fever of 39 degrees Celsius, oral, one hour prior to arrival. The patient was born in Cuba but had resided in South Florida for over 10 years. The patient reported multiple streptococcal pharyngitis infections in the past, the most recent 12 years ago, which were not always treated due to failure to seek medical attention and a reported allergy to penicillin described as a rash without anaphylaxis.

Initial vital signs in the emergency department revealed blood pressure of 112/69 millimeters (mm) of mercury, pulse of 95 beats per minute, respiratory rate of 18 breaths per minute, peripheral capillary oxygen saturation of 99% on room air, and oral temperature of 37.1 degrees Celsius. The patient was alert and oriented to time, place, self, and condition. Physical examination was remarkable for pharyngeal erythema and bilateral tonsillar exudates. The uvula was midline and inflamed. The mucous membranes were moist, and there was no appreciable anterior or posterior lymphadenopathy. The external auditory canals and tympanic membranes were unremarkable. Fluid-filled vesicular lesions were visualized over the right upper eyelid but were absent over the tip, side, and root of the nose. There was no evidence of anterior uveitis, punctate keratitis, or pseudodendrites on the ocular exam. The remainder of the skin examination was free of rashes, and no subcutaneous nodules were identified on the extensor surfaces or along the spinous processes of the thoracic and lumbar vertebrae. The left wrist was swollen but demonstrated no erythema or hyperemia. Left wrist pain was elicited with flexion and extension; however, the range of motion was not reduced. Capillary refill across all digits was normal. The heart rate and rhythm were normal, and there were no rubs, gallops, or murmurs appreciated. Lung sounds were clear to auscultation in all fields. The abdomen was soft, non-tender, and non-distended, and no palpable masses were identified.

The rapid GAS/*Streptococcus pyogenes* test was positive. The complete blood count revealed leukocytosis (white blood cell count: 16.3 × 1,000/microliter (µL) (3.5-10.0 × 1,000/µL)) with neutrophil predominance (absolute neutrophil count: 11.91 × 1,000/µL (2.00-7.15 × 1,000/µL)). The erythrocyte sedimentation rate (ESR) and C-reactive protein (CRP) levels were elevated (61 mm/hour (mm/hr) (0-20 mm/hr) and 18.45 mg/deciliter (mg/dL) (<1.00 mg/dL), respectively). The troponin-I level was 2.796 nanograms/milliliter (ng/mL) (0.000-0.034 ng/mL). The basic metabolic panel was unremarkable, and the mononucleosis test was negative. The electrocardiogram demonstrated a normal sinus rhythm with a ventricular rate of 83 beats per minute, and the PR interval was 138 milliseconds (ms). A swab of the lesions over the right eyelid was collected, and the polymerase chain reaction resulted positive for herpes simplex virus type 1 and negative for herpes simplex virus 2 (Table 1).

**Table 1:** Summary of pertinent laboratory findings GAS: group A *Streptococcus*; µL: microliter; mm: millimeter; hr: hour; mg: milligram; dL: deciliter; ng: nanogram; mL: milliliter HSV: herpes simplex virus; PCR: polymerase chain reaction

Parameter	Patient value	Normal/reference range
Rapid GAS test	Positive	Negative
White blood cell	16.3 × 1,000/µL	3.5-10 × 1,000/µL
Absolute neutrophil count	11.91 × 1,000/µL	2.00-7.15 × 1,000/µL
Erythrocyte sedimentation rate	61 mm/hr	0-20 mm/hr
C-reactive protein	18.45 mg/dL	<1.00 mg/dL
Troponin-I	2.796 ng/mL	<0.034 ng/mL
Mononucleosis test	Negative	Negative
HSV-1 PCR	Positive	Negative
HSV-2 PCR	Negative	Negative

The patient was administered 500 mg azithromycin PO, 10 mg dexamethasone intravenous, 500 mg naproxen PO, and 400 mg acyclovir PO. Given the presence of two major (carditis and monoarthritis) and two minor (fever and elevated ESR and CRP) criteria, ARF was suspected, and the patient was admitted due to concerns of carditis and possible RHD. An echocardiogram with Doppler revealed no significant valvular stenosis or regurgitation or evidence of rheumatic valvular disease. Serial troponin-I levels decreased to 2.622 ng/dL on hospital day 1 and 0.332 on hospital day 2. An infectious disease consult was placed, and it was recommended that the patient continue azithromycin 500 mg PO daily for five days followed by azithromycin 250 mg PO daily for 10 years and valacyclovir 500 mg TID for 14 days. The patient was advised to schedule an outpatient follow-up with infectious disease upon discharge from the hospital. Upon review of the medical record, there were no documented return visits to the healthcare system within 60 days of the hospital discharge date.

## Discussion

ARF is a systemic inflammatory disease that typically follows an untreated or inadequately treated GAS infection commonly presenting as pharyngitis or impetigo. Although the incidence of ARF has decreased since the early 1900s, it remains a significant cause of global morbidity and mortality, particularly in developing nations [10]. The global incidence of ARF is eight to 51 cases per 100,000 people, and the highest incidence is in children between the ages of six and 12 years [5]. ARF is rare in the US due to improved living conditions and the widespread use of antibiotics. However, it continues to be a significant public health concern worldwide, especially in low- and middle-income countries, particularly in regions of Latin America, Africa, and Asia. Certain populations in the US, such as immigrants from endemic regions and Indigenous communities, remain vulnerable, suggesting that socioeconomic factors and limited access to healthcare are significant determinants [10].

The pathogenesis of ARF involves molecular mimicry in which the immune response to GAS antigens cross-reacts with human tissues, particularly in the heart, joints, central nervous system, and skin. This mechanism is believed to be mediated by M-protein, a major virulence factor of GAS, which shares structural homology with myosin in cardiac tissue and other human proteins. The aberrant immune response results in tissue inflammation and damage, leading to the hallmark manifestations of ARF. Environmental factors, genetic predisposition, and virulence of the GAS strain further influence the likelihood of developing ARF [11].

The diagnostic criteria for ARF have evolved over the years. William Cheadle first described the symptoms of carditis, erythema marginatum, polyarthritis, and subcutaneous nodules in 1898 [12]. In 1944, Thomas Duckett Jones delineated the first set of diagnostic criteria known as the Jones criteria. These criteria were modified by the American Heart Association (AHA) in 1992 and further revised in 2015 to the current version [13]. The revised Jones criteria are divided into two main categories, major and minor. Major criteria consist of arthritis, carditis, subcutaneous nodules, erythema marginatum, and chorea, while the minor criteria include fever, elevated ESR/CRP, and a prolonged PR interval. To diagnose ARF, there must be evidence of a positive throat culture for GAS or elevated antistreptolysin O titers. Carditis is the most commonly presenting major criterion occurring in 50%-70% of first episodes of ARF and can involve pancarditis and valvulitis. The AHA guidelines emphasize the use of echocardiography/Doppler studies to detect subclinical carditis, which is defined as valvulitis (mitral or aortic regurgitation) without findings during cardiac auscultation. Arthritis typically presents as a migratory polyarthritis affecting large joints such as the knees, ankles, elbows, and wrists and is seen in 35%-66% of cases. Chorea, a neurological manifestation, occurs in 10%-30% of cases and is more common in female patients. Subcutaneous nodules and erythema marginatum are less common but highly specific, occurring in less than 10% and 6% of cases, respectively. RHD is primarily diagnosed through echocardiography, and the American Society of Echocardiography recommends the use of echocardiography to identify typical valvular and subvalvular abnormalities such as commissural fusion, leaflet thickening, and restricted leaflet mobility and to assess their impact on hemodynamics and pulmonary artery pressures [14].

ARF shares overlapping clinical features with a range of conditions and requires careful differentiation to establish an accurate diagnosis. Post-streptococcal reactive arthritis is a common differential, presenting with joint inflammation following a GAS infection, but lacks the systemic manifestations of ARF [15]. Infective endocarditis may also mimic ARF in patients with fever and heart murmurs; however, positive blood cultures and evidence of vegetations on echocardiography are more suggestive of endocarditis than ARF [16]. Juvenile idiopathic arthritis, particularly the polyarticular subtype, resembles ARF’s migratory arthritis but does not involve carditis or follows a GAS infection [10]. Systemic lupus erythematosus can mimic ARF due to its multisystem involvement, including arthritis, rash, and cardiac manifestations, but it is distinguished through autoantibody profiles and the absence of a GAS trigger. Additionally, Kawasaki disease, which presents with fever, rash, and coronary artery involvement, should be considered in young children, though its mucocutaneous signs differentiate it from ARF [17].

Therapeutic management of ARF and RHD focuses on treating the acute infection, providing secondary prophylaxis, and mitigating complications. The primary treatment goal is to eradicate the GAS infection, and penicillin is considered the first line. For patients allergic to penicillin, alternatives such as azithromycin or erythromycin can be used [18]. Non-steroidal anti-inflammatory drugs (NSAIDs) like aspirin or naproxen are used to reduce inflammation and relieve symptoms, and, in severe cases, corticosteroids may be considered. The American College of Cardiology and AHA recommend long-term antibiotic prophylaxis to prevent recurrent ARF and progression to RHD. Benzathine penicillin G is administered intramuscularly every three to four weeks. Alternatives include oral penicillin V, sulfadiazine, and azithromycin for those allergic to penicillin [18]. The duration of prophylaxis is dependent on the presence and severity of carditis. Patients with carditis and evidence of residual heart disease should continue prophylaxis treatment for at least 10 years or until age 40, whichever is longer, while those with carditis and no evidence of residual heart disease should continue prophylaxis treatment for at least 10 years or until age 21, whichever is longer. If the patient demonstrates no evidence of carditis, prophylaxis should continue for five years or until age 21, whichever is longer [18]. For severe RHD manifesting as acute heart failure, standard management including diuretics, angiotensin-converting enzyme inhibitors, and beta-blockers should be employed, and valve repair or replacement surgery should be considered on a case-by-case basis [19].

## Conclusions

We discussed an atypical presentation of ARF in an adult patient residing in the US presenting with fever, sore throat, left wrist arthralgia, and chest pain. It is important to consider the possibility of ARF in all patients being worked up or treated for GAS pharyngitis, and, if clinical suspicion warrants, the history, physical examination, and diagnostic testing should include sufficient investigation for the presence of revised Jones major and minor criteria. Therapeutic management involves eradicating the acute infection with antibiotics, and cephalosporins, azithromycin, or erythromycin should be administered if the patient has a verified penicillin allergy. Further studies including echocardiography should be pursued if carditis is suspected to assess for the presence of RHD as findings will influence the duration of therapeutic and secondary prophylactic treatment.
